# Monthly Summary

**Published:** 1879-12

**Authors:** 


### MONTHLY SUMMARY.

*Artificial Palate.*—Dr. Erastus Olin writes: W. F.———, Act, 15, consulted me, in Dec., 1873, in reference to a cleft in his mouth, extending through the hard and soft palate and alveolar ridge. There was an overlap on one side of the cleft only, the hair lip having been treated early in life.

To be brief, only three natural teeth have made their appearance in the superior maxillary, namely: two molars on the right side, and one on the left. Moreover, there is neither vomer, uvula, or palato-pharyngeus. I therefore made an ordinary upper denture of seven teeth, and continued backwards from its posterior border, an artificial velum of elastic rubber. To my surprise, the voice of the patient was quickly changed from a nasal twang to a bass key.

These cases require great pains-taking. Otherwise, failure will be the result.

*The Relation between the Age of the Mother and the Sex of the Child.*—Dr. Bidder, in the *Zeitschrift fur Geburtshulfe und Gynakologie*, Band 2, gives the results of his observations in this subject. In 4441 primiparæ the proportion was 100 female to 111.5 male children. Very young primiparæ gave birth to a large proportion of boys; those of twenty or twenty-one years old had more girls than boys: while as the age increased the proportion of male children again arose. In 7430 multiparæ there were 100 female to 112.4 male children. The proportion of males exceeded that of females in those aged from seventeen to twenty-one; the number sank in the twenty-second and twenty-third years, reached its minimum at the ages of twenty-four and twenty-five, and then again increased in proportion to the age of the mothers.—*Med. and Surg. Reporter.*

*Steel Rust.*—According to the *Chemiker Zeitung*, articles of steel which have become rusty may be cleansed by brushing with a paste made up of 30 parts of cyanide of potassium, 30 parts curd soap, 60 parts of precipitated chalk, and a sufficiency of water. Great care is required in preparing and using this poisonous mixture.

*Nickel Plating Without a Battery.*—The *Engineer* publishes Prof. Slatba's new unpatented process for nickel plating in the wet way without the use of a battery: To a dilute solution of chloride of zinc (five to ten per cent.) enough nickel sulphate is to be added to impart a decidedly green color to it, and the solution is then to be heated to boiling in a porcelain vessel. The clouding of the liquid from the separation of a basic zinc salt need not be heeded, as it will not interfere with the effectiveness of the bath. The articles to be nickel plated are first freed from oxide or grease, and then suspended in the solution from thirty to sixty minutes, the bath being kept at a boiling temperature. When the articles are observed to be uniformly coated they may be removed, washed in water, in which a little chalk is suspended, dried, and finally polished with chalk or other suitable material. By the substitution of a cobalt salt instead of nickel, objects may be similarly coated with cobal.—*Pharmacist and Chemist.*

---

*Tobacco Smoke.*—The authorities of several German cities, says *Chambers' Journal*, have been seriously considering the evils resulting from smoking, now so generally practiced by boys. In certain towns, the police have been ordered to forbid all boys under sixteen to smoke in the streets, and a punishment by fine or imprisonment is meted to offenders. It has been the testimony of several eminent physicians that the too general and excessive use of tobacco is the main cause of color blindness, now occasioning such great anxiety from its influence upon railway and other accidents, and also upon military efficiency.—*Pacific Med. and Surg. Journal.*

---

*Sugar and Teeth.*—The *Medical Journal* of Charleston, S. C., thus stated the conclusions of M. Lerez: "1st. Refined sugar injures teeth—either by immediate contact, or by gas developed in the stomach. 2nd. That a tooth soaked in sugar-water becomes jelly-like, from the sugar combining with the lime of the tooth."—*Dental News.*

---

*Artificial India-Rubber.*—A mass said to resemble India-rubber and soluble in linseed oil, may be prepared by heating in an iron kettle, which should be only half filled, ten pounds of sulphur and twenty pounds of rapeseed oil, with constant stirring until the sulphur has been melted and the mass begins to swell; then immediately pouring it into a mould, dusted with some kind of powder, or upon a slab moistened with water, when it will harden at once. Linseed oil may take the place of rapeseed, when less sulphur must be used.—*Druggists Circular.*

